# Ultra-Processed Food Consumption Patterns and Their Association with Blood Pressure Among Young Adults: A Cross-Sectional Study

**DOI:** 10.3390/nu18101617

**Published:** 2026-05-20

**Authors:** Karthikeyan Ramanujam, Abhigna Mahathi, Jarupula Namrathaa Pawar, Maheshwari Matla, Harichandana Ponnapalli, Vinay Kumar Soma, Keerthana Gajjala, SuryaGoud S. Chukkala, Mahesh Kumar Mummadi, SubbaRao M. Gavaravarapu, G Bhanuprakash Reddy, Jagajeevan Babu Geddam, Samarasimha Nusi Reddy

**Affiliations:** ICMR-National Institute of Nutrition, Hyderabad 500007, India; ramanujam.k@icmr.gov.in (K.R.); mahathiprasad302@gmail.com (A.M.); namrathaapawar@gmail.com (J.N.P.); m.maheshwari2k2@gmail.com (M.M.); chandanaponnapalli@gmail.com (H.P.); vinaysoma100@gmail.com (V.K.S.); gajjalakeerthana28@gmail.com (K.G.); suryachuka@gmail.com (S.S.C.); mahidoc@yahoo.com (M.K.M.); subbarao.gm@icmr.gov.in (S.M.G.);

**Keywords:** ultra-processed foods, hypertension, sodium intake, young adults, college students

## Abstract

**Background:** Hypertension is being increasingly observed among young adults in urban India, alongside rapid dietary transitions and rising consumption of ultra-processed foods (UPFs). The current study aimed to assess the frequency and patterns of UPF consumption and examine their association with high blood pressure among urban college students. **Methods:** A cross-sectional study was conducted among 311 undergraduate students aged 18–24 years from three colleges in Hyderabad, India. Our study used a validated automated device to measure blood pressure. Dietary intake over the previous month was assessed using a 24-item food frequency questionnaire capturing commonly consumed UPFs. After adjusting for age, sex, and socioeconomic variables, multivariable logistic regression was performed to assess the relationships between UPF consumption categories and high blood pressure. Ninety-five percent confidence intervals (CIs) for adjusted odds ratios (AORs) were reported. **Results:** Overall, 12.5% of participants had high BP (≥140/90 mmHg). The prevalence was higher among males and those aged >20 years. In the adjusted analyses, males had significantly higher odds of having high BP (AOR: 4.96; 95% CI: 1.64–15.01), as did students from higher-income households (AOR: 3.22; 95% CI: 1.07–9.66). Consumption of high-fat and/or high-salt UPFs at or above the median was independently associated with high BP (AOR: 2.85; 95% CI: 1.16–6.99). Taste, availability, and low cost were common drivers of UPF intake. **Conclusions:** Higher consumption of high-fat and/or high-salt ultra-processed foods was associated with higher odds of elevated blood pressure among urban young adults. These findings warrant further longitudinal investigation and may help inform the development of targeted dietary awareness and food environment interventions in college settings.

## 1. Introduction

Globally, hypertension is a major risk factor for cardiovascular diseases and early death [1]. According to the World Health Organization, nearly 1.4 billion adults are living with high blood pressure around the world, with a substantial proportion remaining undiagnosed or inadequately controlled [2]. Over 10.8 million fatalities and roughly 9% of all DALYs in 2019 were caused by increased systolic blood pressure, the leading cause of death worldwide [3]. Due to its high incidence and sizable population, India alone is home to more than one-fifth of the world’s hypertensive population [4]. Hypertension has historically been considered a disease of middle and older age populations; however, studies in recent years have reported a rising prevalence among younger populations, including those in college settings [5,6,7,8]. Early onset of hypertension increases the lifetime risk of cardiovascular diseases, emphasizing the importance of identifying modifiable lifestyle and dietary risk factors during young adulthood [9]. In India, rapid urbanization and associated lifestyle changes have been accompanied by a rising burden of hypertension among younger populations. Studies from urban settings reported a notable presence of prehypertension and hypertension among college-going young adults, often related to unhealthy dietary practices, sedentary behaviour, excess body weight, and family history of cardiometabolic diseases [10,11]. These findings highlight young adulthood as a critical window for preventive interventions, particularly in urban environments where dietary patterns are undergoing marked transformation.

In parallel with this rising burden, young adults in urban India are experiencing notable shifts in dietary patterns, characterized by increased consumption of energy-dense, convenience foods and a decline in traditional home-prepared diets. Dietary patterns among young adults have changed substantially in recent decades, largely driven by urbanization, globalization, and the expansion of commercial food systems [12]. A key feature of this shift is the increasing consumption of UPFs. UPFs are industrial formulations predominantly made from substances extracted or derived from foods, often containing little or no intact whole foods. These foods include additives such as colourants, flavourings, emulsifiers, and preservatives that enhance palatability and shelf-life [13]. UPFs are the highest level of industrial processing in the NOVA categorization system, which groups foods according to the degree of processing [13].

In the Indian context, UPF consumption has increased considerably over the past decade, reflecting broader nutrition transitions associated with urbanization and changing food environments. Although nationally representative estimates remain limited, the available evidence and market trends suggest growing consumption of products such as packaged snacks, sugar-sweetened beverages, biscuits, instant noodles, and ready-to-eat foods, particularly among urban populations and young adults. These products are widely accessible and often preferred due to their convenience, affordability, and taste, indicating an increasing integration of UPFs into daily diets [14,15].

Due to aggressive marketing, price, convenience, and the growth of urban food environments, UPF availability and consumption have expanded dramatically, especially among children, adolescents, and young people [16]. These foods are often low in dietary fibre and micronutrients and high in sodium, sugar, and harmful fats, which can raise blood pressure [17]. Diets high in UPFs are characterized by elevated sodium intake, a key determinant of elevated blood pressure. High salt intake contributes to increased blood pressure mostly through volume expansion and vascular alterations. Excess salt intake leads to water retention, increasing intravascular volume and arterial flow, which raises blood pressure [18].

Recent epidemiological studies showed that UPF consumption led to an increased risk of hypertension and other adverse cardiovascular diseases [19]. Cohort studies and meta-analyses suggest that higher UPF consumption tends to lead to elevated blood pressure levels and a greater risk of developing hypertension in adult populations [19,20,21]. Among college students, UPF consumption is influenced by sociocultural and environmental factors, including peer norms and the college food environment [22]. Differences in socioeconomic background and parental education also shape consumption patterns and should be considered when examining dietary associations [23]. However, much of this evidence comes from high-income countries, and data from low- and middle-income settings, particularly among young adults, remain limited. Evaluating consumption frequency provides insight into the behavioural drivers of diet-related health risks and can inform nutrition education interventions.

India is undergoing a nutrition transition characterized by rising UPF consumption [24]. Urban college students are particularly susceptible to high UPF exposure due to their immersion in evolving food environments. Despite rising UPF consumption in India, evidence linking domain-specific UPF intake, particularly high-salt and high-fat UPFs, with blood pressure among young adults remains limited. This study contributes to the existing literature by focusing on urban Indian young adults, a population undergoing rapid dietary transition but are underrepresented in current evidence. It incorporates domain-specific categorization of ultra-processed foods based on nutrient profiles (e.g., high salt, fat, and sugar combinations) and examines both consumption patterns and underlying drivers of intake. Therefore, this study aimed to examine the frequency and patterns of UPF consumption and assess their association with high blood pressure among urban college students in Hyderabad, India.

## 2. Methods

### 2.1. Sample Size

The sample size for this study was determined using a hypertension prevalence of 7.6% reported in previous studies and systematic reviews, with an absolute precision of 3% and a 95% confidence level [25,26]. Based on the assumptions, the minimum required sample size was estimated to be 293 participants. To account for an anticipated 10% non-response rate, the sample size was inflated, resulting in a final calculated sample size of 309 participants. In this study, 311 participants were recruited and included in the analysis.

### 2.2. Study Population and Settings

A cross-sectional study was conducted between September and October 2025 among undergraduate students aged 18–24 years from three colleges affiliated with a public university in Tarnaka, Hyderabad. The sampling frame was created using official enrolment records provided by the participating institutions. A final sample of 311 students was included in the analysis, which included participants selected by simple random sampling from the aggregate student list acquired from all three colleges. Students enrolled in the undergraduate programme of the selected colleges were eligible to participate if they were between 18 and 24 years of age, free from any known chronic or acute health conditions, not diagnosed with cardiovascular disease, not on antihypertensive medications, and willing to participate by providing written informed consent.

### 2.3. Data Collection Tools

A structured, pre-tested questionnaire was developed specifically for this study. Prior to the primary study, students from the chosen colleges participated in a pilot study to determine which processed and ultra-processed foods are often consumed. Although formal validation was not performed, efforts were made to enhance content validity through pilot testing and expert review. Based on the pilot assessment, commonly consumed processed food items were mapped to the NOVA food classification framework, and those categorized as ultra-processed foods were incorporated into the final questionnaire for dietary assessment [27]. The questionnaire was divided into two sections that were intended to collect detailed background and dietary data. To provide context for interpreting dietary patterns, the first section gathered sociodemographic information such as age, sex, academic stream, year of study, type of residence, and family income. The second section assessed dietary intake using the Food Frequency Questionnaire (FFQ) [14,28,29], which included a total of 24 commonly consumed ultra-processed food items comprising biscuits, bread, packed breakfast cereals (oats, cornflakes, muesli), bun, cakes and cake mixes, carbonated beverages, chocolates, cookies, energy drinks, energy/protein bars, flavoured yoghurt, frozen foods with additives, cheese, protein powders, fruit juices (tetra packs), ice creams, jams, instant noodles, mayonnaise, other instant foods with additives, pastries, sauces, savoury and sweet packaged foods, and spreads, and was used to record the frequency of consumption (daily, thrice a week, twice a week, once a week, once in 15 days, once a month, rarely and never) of each item over the preceding one month. The full FFQ used in this study is provided in the Supplementary Materials (Supplementary Table S3). All the data were captured electronically in real time using KoboToolbox [30].

### 2.4. Blood Pressure Measurement

An automated, verified digital sphygmomanometer (Omron HEM-8712; Omron Healthcare Co., Kyoto, Japan) was used to take blood pressure readings under controlled circumstances. Each participant was seated comfortably prior to measurement, with the cuff positioned on the left arm at heart level. To reduce the variability, three readings were taken at five-minute intervals, and the average was used for analysis. To minimize external stressors that could affect the results, measurements were taken in a quiet room. For the analysis, blood pressure values were categorized as follows: Normal (<120/80 mmHg), Elevated (systolic blood pressure (SBP) of 120–129 mmHg and diastolic blood pressure (DBP) < 80 mmHg), High BP stage 1 (SBP of 130–139 mmHg or DBP of 80–89 mmHg) and High BP stage 2 (SBP ≥ 140 mmHg or DBP ≥ 90 mmHg). High blood pressure was defined as SBP ≥ 140 mm Hg or diastolic blood pressure (DBP) ≥ 90 mm Hg according to American Heart Association (AHA, 2017) guidelines [31]. Blood pressure categories were defined based on measured values using standard cut-offs as per established guidelines. These measurements were used to classify participants as having elevated blood pressure for epidemiological analysis and do not constitute a clinical diagnosis of hypertension, which requires repeated measurements on separate occasions.

### 2.5. Ethics Approval and Consent

The Institutional Ethics Committee of the National Institute of Nutrition (ICMR) reviewed and approved the study protocol (Protocol No. NIN/IEC/2025/8/ER/AN/13). Following ethical clearance, data collection was initiated. Before participation, all students were informed about the objectives, purpose, and scope of the study, and the participants’ written informed consent was obtained. Students were informed that participation in the study was completely voluntary and that they can leave at any time without facing any consequences. Confidentiality of the participant’s information was strictly maintained throughout the study. Students identified with high blood pressure during the study were counselled and referred to nearby healthcare facilities for further evaluation and management. All study procedures involving participants were conducted in accordance with the principles outlined in the 1964 Declaration of Helsinki and its subsequent amendments.

### 2.6. Statistical Analysis

Descriptive statistics were used to summarize the characteristics of the study participants and variables. SBP and DBP were summarized as continuous variables using means and standard deviations; frequencies and percentages were used for categorical variables. The distribution of total scores was used to derive the median (50th percentile), which served as the cut-off to classify participants into lower and higher UPF consumption groups. In addition to the overall UPF score, domain-specific UPF categories were developed based on nutrient thresholds derived from the Indian Dietary Guidelines (DGI) [32]. Information on sugar, sodium, and fat contents was obtained from food labels and supplemented with values from the USDA Food Composition Database (Supplementary Table S1). Using these criteria, UPFs were classified into high-salt, high-fat, high-fat and salt, high-sugar, high-sugar and salt, and high-sugar and fat food groups. Domain-specific consumption scores were created by adding the frequency scores for each category’s items, and participants were divided into higher and lower intake groups for further analysis using median cut-offs. The Shannon diversity index was calculated as a continuous measure to quantify the diversity of UPF consumption for each participant based on the relative frequency of consumption of individual UPF items. In the primary analysis, the index was retained as a continuous variable to preserve variability and statistical power. For descriptive purposes, it was additionally categorized based on the median (50th percentile) to facilitate comparisons between lower and higher diversity groups [33] and examined for association with blood pressure outcomes and hypertension. An UpSet plot was employed to evaluate the distribution and co-occurrence of self-reported reasons for ultra-processed food consumption and their association with mean UPF scores [34]. The plot summarizes the frequency of individual reasons and their intersections, allowing for the assessment of combinations of reasons corresponding to higher levels of UPF intake.

Additionally, to evaluate the degree of correlation between blood pressure status and the gathered covariates, univariable and multivariable logistic regression analyses were performed. The final multivariable logistic regression model contained variables that showed statistical importance in the univariable analysis as well as those deemed theoretically and culturally significant. The associations were quantified by estimating crude odds ratios, adjusted odds ratios (ORs), and 95% confidence intervals (CIs). Statistical significance was defined as a *p*-value of less than 0.05. The model’s goodness-of-fit was evaluated using the Hosmer–Lemeshow test. Analyses were based on complete cases, and no imputation of missing data was done. Stata version 18.0 (StataCorp LLC, College Station, TX, USA) was used for statistical analyses.

## 3. Results

Of the 311 participants, 68.0% were under 20 years old, and 64.0% were male. Individuals over 20 had a higher mean systolic blood pressure (SBP) and diastolic blood pressure (DBP) (116.3/79.1 mmHg) compared to those under 20 (109.5/75.0 mmHg), and the prevalence of high blood pressure was higher (19.2% vs. 9.4%). Males exhibited a substantially higher prevalence of high blood pressure (17.6% vs. 3.5%) and a higher mean SBP and DBP (118.0/79.0 mmHg) than females (99.8/71.6 mmHg). The prevalence of high blood pressure was slightly higher among participants living in hostels or other accommodations (13.7%) than among those living at home (12.3%) (Table 1).

Students in the third and fourth years of undergraduate study had a higher mean SBP and DBP (114.0/78.9 mmHg) and a higher prevalence of high blood pressure (18.7%) compared with first- and second-year students (9.7%). Participants from households with a monthly family income of >₹100,000 had a higher mean SBP and DBP (114.6/80.3 mmHg) and a notably higher prevalence of high blood pressure (31.6%) than those from lower-income households (11.3%). Variations were also observed across parental education and occupation, with generally higher mean blood pressure among participants whose parents had higher educational attainment or were employed in government or private sectors. However, these patterns were not uniform across all categories (Table 1).

### 3.1. Prevalence of High Blood Pressure

The distribution of blood pressure categories by age and sex is presented in Table 2. Of those under 20, 61.8% (95% CI: 55.1–68.3) had normal blood pressure, 5.7% (3.2–9.4) had high blood pressure, 23.1% (17.8–29.1) had stage 1 hypertension, and 9.4% (6.1–14.0) had stage 2 hypertension. On the other hand, regarding participants over 20 years old, there were larger proportions of participants with stage 1 (29.3%; 21.3–38.8) and stage 2 hypertension (19.2%; 12.3–27.8) and a smaller proportion with normal blood pressure (44.4%; 34.9–54.3), suggesting a shift toward higher blood pressure categories with advancing age.

According to the analysis based on sex, 42.9% (36.2–49.9) of males had normal blood pressure, while 8.5% (5.2–13.1) had elevated blood pressure, 30.8% (24.6–37.5) had stage 1 hypertension, and 17.7% had stage 2 hypertension. Among females, there was a higher proportion with normal blood pressure and substantially lower proportions in the elevated and hypertensive categories overall.

### 3.2. UPF Consumption Pattern

**Figure 1:**
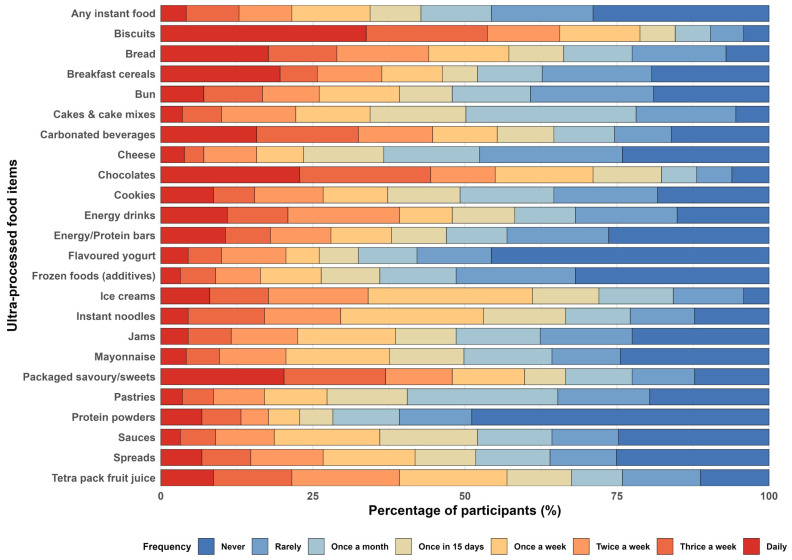
Frequency distribution of consumption of UPFs among study participants.

Figure 1 shows the distribution of ultra-processed food (UPF) consumption across frequency categories. Biscuits, chocolates, and packaged savoury/sweet foods were the most frequently consumed items, with a considerable proportion reporting intake three times weekly or daily. Bread and breakfast cereals were also commonly consumed, primarily weekly or more frequently. In contrast, protein powders and energy drinks were reported as rarely or never consumed. Cheese and mayonnaise showed low regular intake. Carbonated beverages, ice creams, pastries, frozen foods, jams, and other instant foods were mostly consumed occasionally (once weekly or fortnightly), with limited daily use. Overall, the pattern reflects frequent intake of snack-based UPFs and intermittent consumption of other categories.

Table 3 shows that consumption of combined high-fat and high-salt UPFs was significantly associated with high blood pressure, with a greater proportion of hypertensive participants reporting intake ≥ median than normotensive individuals (71.8% vs. 53.3%; *p* = 0.030). A stronger association was observed for intake of any high-fat and/or high-salt foods (76.9% vs. 50.7%; *p* = 0.002). Consumption of high-sugar and high-salt foods showed a borderline association (66.7% vs. 50.0%; *p* = 0.051). Although higher consumption of high-fat foods alone was more common among those with high BP (71.8% vs. 57.4%), the association was not statistically significant (*p* = 0.086). High-salt foods alone, high-sugar foods, high-sugar and fat foods, or UPF dietary diversity did not significant correlations (all *p* > 0.05). However, a greater proportion of people with high BP was observed in the high-diversity group.

**Figure 2:**
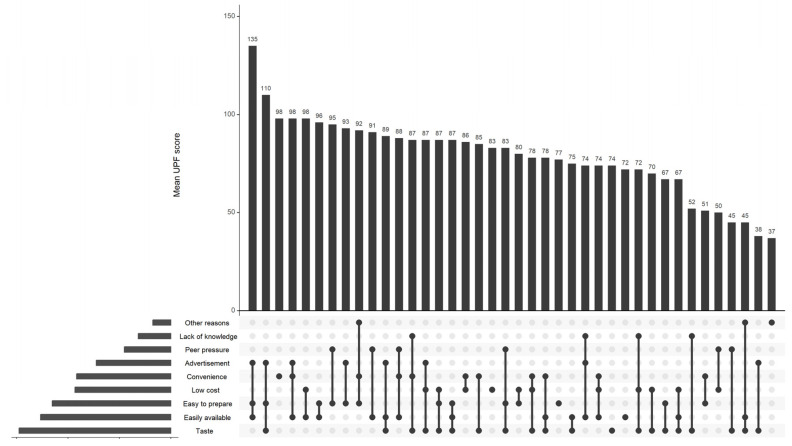
Reported reasons for consumption of UPFs and mean intake score among young adults. Each dot represents the presence of a specific reason for ultra-processed food consumption within a given combination, and vertical lines indicate reasons that co-occur. The bars in the upper panel represent the mean UPF intake score of participants reporting each combination of reasons. The horizontal bars on the left represent the number of participants reporting each reason for consuming ultra-processed foods.

Figure 2 shows the combinations of reported reasons for UPF consumption and the corresponding mean UPF consumption scores. Taste was the most frequently reported reason for consuming ultra-processed foods, as reflected by the largest horizontal bar on the left. Easy availability, ease of preparation, and low cost were also commonly cited, indicating that both sensory appeal and convenience-related factors play a major role in UPF consumption among young adults.

The intersection bars in the top panel show that multi-factor motivations were more common than single reasons. The highest mean UPF scores were observed for combinations involving easy availability, convenience, advertisement exposure, and ease of preparation, either alone or in combination, indicating that environmental and accessibility-related factors have a strong link with higher UPF consumption. On the other hand, combinations involving taste alone or ignorance tended to display relatively lower mean UPF scores, indicating that these factors are linked to more moderate UPF intake when reported separately.

Less commonly reported reasons, such as peer pressure, advertising, and a lack of knowledge about healthier alternatives, appeared mainly in combination with more dominant factors, such as taste or convenience.

Supplementary Figure S1 illustrates the association between ultra-processed food (UPF) score and blood pressure among participants. A modest but statistically significant positive association was observed between UPF score and systolic blood pressure (β = 0.06, *p* = 0.037), indicating that higher UPF consumption is associated with a slight increase in systolic BP in panel A, whereas panel B shows a significant positive association between UPF score and diastolic blood pressure (β = 0.05, *p* = 0.0069). Although the effect sizes are small, the consistent upward trend across both panels suggests that greater ultra-processed food consumption is associated with higher blood pressure.

In Table 4, participants aged over 20 years had higher odds of high blood pressure compared with those aged 20 years or younger (OR: 2.28; *p* = 0.018); however, this association did not remain statistically significantly after adjustment for other covariates (AOR: 1.320; 95% CI: 0.54–3.19; *p* = 0.538). Male participants had substantially higher odds of high blood pressure than females in both the unadjusted (OR: 5.85; *p* = 0.001) and adjusted analyses (AOR: 4.96; 95% CI: 1.64–15.01; *p* = 0.005).

Participants from households with a higher monthly income (>₹100,000) were more likely to have high blood pressure compared with those from lower-income households. This association remained significant after adjustment (AOR: 3.22; 95% CI: 1.07–9.66; *p* = 0.037). Year of study was associated with high blood pressure in the univariable analysis, with students in the third and fourth years of undergraduate study showing higher odds than those in the first and second years (OR: 2.13; *p* = 0.030); However, this variable was not included in the final multivariable model due to its collinearity with age, as both variables capture a similar underlying effect.

Among dietary factors, participants consuming any high-fat and/or high-salt foods at or above the median had significantly higher odds of high blood pressure in both the crude (OR: 3.23; *p* = 0.003) and adjusted analyses (AOR: 2.85; 95% CI: 1.16–6.99; *p* = 0.022), indicating an independent association even after controlling for potential confounders. In contrast, high-salt and sugar food consumption showed only a borderline association in the crude model (OR: 2.01; *p* = 0.055) and was not statistically significant after adjustment (AOR: 1.25; 95% CI: 0.53–2.91; *p* = 0.604), suggesting that other covariates may partly explain the observed crude association.

## 4. Discussion

The present study found that consumption of ultra-processed foods with a high fat and/or high salt content was independently associated with elevated blood pressure among young adults, even after adjusting for sociodemographic factors. This extends the prior Indian evidence, including urban cohort studies reporting associations between packaged savoury snack intake and hypertension risk by isolating specific UPF subcategories rather than examining total UPF intake alone, and by demonstrating this relationship in a college-going population that has been underrepresented in domestic research. Internationally, studies from Brazil [35,36], Spain [37], and the United States [38] have similarly identified high-fat and high-salt UPF categories as more strongly predictive of elevated blood pressure than overall UPF diversity or total energy intake from processed foods. Unlike several of these international studies, which were conducted in middle-aged or mixed-age populations, our findings establish that these subcategory-specific effects are already detectable in young adulthood, a period when hypertension is frequently unrecognized and unmanaged. In contrast, foods high in both salt and sugar did not retain significance after adjustment, and UPF diversity showed no independent association, suggesting that the type and frequency of specific UPF categories rather than dietary variety are the more relevant determinants of early hypertension risk [17,39,40,41].

The study’s findings indicate that individuals aged 20 or older and males had a higher prevalence of high blood pressure. This age-related pattern, which most likely implies persistent behavioural and metabolic exposures, is consistent with NFHS-5 findings that demonstrate a constant rise in hypertension across older adolescent and young adult groups [42,43]. After multivariable adjustment, the association between age and high blood pressure became less significant, suggesting that age-related variation may be largely due to correlated dietary and lifestyle factors rather than an independent effect of age [17]. Several studies, including some Indian studies, suggest that high blood pressure in men may be attributed to excessive salt consumption and unfavourable lifestyle habits [9], whereas the lower prevalence of high blood pressure among females may be explained by sex-hormone-related vascular protection [9,44]. This is further substantiated by a few studies indicating that male students frequently rely on external food sources, whereas female students are more inclined to prepare their own meals and include fruits and vegetables in their diets [28]. Individuals residing in hostels exhibited a slightly elevated prevalence of high blood pressure compared to those living at home, possibly due to a repetitive menu, and those relocating for higher studies often have difficulty in adjusting to new food environments, resulting in a reliance on UPFs to satisfy cravings, resulting in higher blood pressure [28]; however, this correlation did not show statistical significance in the adjusted analyses, potentially indicating that lifestyle differences associated with living outside of the home does not affect UPF consumption. The opposite pattern has been observed in other student populations, where day-scholar residents tend to exhibit a higher prevalence of pre-hypertension compared with adults living in hostels [45,46]. The association between higher income and high blood pressure observed in this study is consistent with evidence from high-income countries showing that socioeconomic advantage may coexist with lifestyle-related hypertension risk, including greater consumption of UPFs and sedentary behaviours [47].

In our study, individuals showed selective consumption of ultra-processed foods, daily consumption of biscuits, chocolates, and packaged snacks, which reflects the particular stage of nutrition transition seen among urban young adults in India. India’s nutrition transition, characterized by a shift away from traditional cereal- and pulse-based diets toward energy-dense, micronutrient-poor processed foods, has markedly accelerated over the past two decades alongside rapid urbanization, rising incomes, and the aggressive expansion of the packaged food industry. Indian young adults appear to be at an intermediate stage, adopting convenient snack-type UPFs while not yet normalizing meal-replacement products, unlike high-income countries. This pattern aligns with findings from the Pune 2024 study [14] and other Indian university-based research [28], which similarly report high intake of sweet snacks and bakery items alongside lower consumption of sugary beverages and fast food, a contrast to the patterns reported in European and North American student populations. These differences likely reflect the interaction of local food environments, price accessibility of certain UPF subcategories, and culturally embedded snacking practices, rather than a uniform global convergence in UPF consumption.

Higher UPF intake was observed when taste coincided with environmental factors such as convenience, availability, low cost, and advertising. This pattern was found to be consistent with international evidence that showed that palatability initiates UPF choice, while food environments characterized by accessibility and convenience reinforce habitual consumption. Similar trends have been reported in an Indian study, particularly in urban populations, where lifestyle changes and increasing availability of ready-to-eat foods have shifted dietary practices [48]. The importance of environmental and structural factors in determining UPF consumption intensity is highlighted by the fact that taste alone or ignorance was linked to significantly lower intake. These results imply that although these factors influence UPF intake, they are secondary rather than primary motivators. These UPF consumption habits may originate in adolescence as most food habits are picked up from that age. Overall, the pattern indicates that structural and environmental factors, including availability and convenience, amplify the influence of taste, reinforcing habitual consumption of UPFs among young adults [49,50].

In addition to dietary factors, behavioural and psychosocial determinants such as psychological stress, academic pressure, and sleep patterns may influence blood pressure among young adults and act as potential confounders. Evidence suggests that chronic stress and poor sleep, both in duration and quality, are associated with an increased risk of hypertension [51,52]. These factors often coexist in college settings and may jointly contribute to elevated blood pressure [53]. As they were not assessed in the present study, residual confounding cannot be ruled out. Future research incorporating these variables would provide a more comprehensive understanding of blood pressure determinants in this population.

These findings highlight the importance of the Dietary Guidelines for Indians [32], which recommends limiting the intake of ultra-processed foods and foods high in salt, sugar, and unhealthy fats (HFSS) to reduce the risk of non-communicable diseases, including hypertension. In line with these recommendations, the results support policy measures, such as a rise in taxation on packaged ultra-processed foods, reduced availability of high-salt items in and around college campuses, and promotion of healthier food alternatives at an affordable price. Increasing awareness and nutritional education programmes in universities and reaching out to parents could help young adults make healthier food choices. Early prevention of hypertension and associated cardiovascular risks may be aided by such integrated fiscal, environmental, and educational strategies. These results highlight the significance of young adulthood as a crucial stage of life when dietary exposures may influence the trajectories of long-term cardiovascular risk.

### Strengths and Limitations

This study has several notable strengths. It focuses on college-going young adults, a population that is underrepresented in Indian dietary and hypertension research despite being at a critical life stage for the development of long-term cardiometabolic risk. Blood pressure was measured using standardized procedures with three readings, reducing measurement error and improving reliability. The study provides a detailed assessment of UPF consumption using a comprehensive FFQ covering multiple UPF groups and a UPF diversity index (Shannon index), which allowed for an evaluation beyond total UPF intake. Analysis of self-reported reasons for UPF consumption further linked behavioural and environmental factors to intake patterns. Adjustment for key sociodemographic variables enabled a clearer assessment of the independent association between high-fat and salty UPF consumption and high blood pressure.

A key limitation of this study is the absence of data on important potential confounding variables, particularly anthropometry, physical activity, psychological stress, academic pressure, and sleep patterns, which were not assessed and therefore could not be accounted for in the analysis. As these factors may influence both dietary behaviours and blood pressure, their omission limits our ability to determine whether the observed association between UPF consumption and elevated blood pressure is independent of obesity and other lifestyle-related factors. Consequently, residual confounding is likely, and the findings should be interpreted with caution.

This study also has certain other limitations that should be considered when interpreting the findings. First, the dietary intake was self-reported, which could result in incorrect consumption frequency classification due to recollection and social desirability biases. In addition, the lack of formal validation may affect measurement accuracy and comparability with other studies. Second, the lack of data on portion sizes made it difficult to determine the precise amounts of ultra-processed foods and nutrients consumed. Third, the assessment included a limited number of ultra-processed food items, selected based on a pilot study conducted in the participating colleges to capture the most frequently consumed foods, which may affect the generalizability of the findings. Furthermore, the cross-sectional design precludes causal inference and does not establish temporality; therefore, reverse causality cannot be ruled out.

## 5. Conclusions

This study found that higher consumption of ultra-processed foods, particularly those high in fat and/or salt, was associated with higher odds of elevated blood pressure among urban young adults. These findings suggest a possible relationship between dietary patterns and blood pressure in this population. However, given the cross-sectional design and the absence of key confounding variables, particularly anthropometry and physical activity, the observed associations should be interpreted with caution and cannot be considered independent or causal.

While causality cannot be established, these findings highlight the need for further research examining dietary patterns in relation to blood pressure in young adults. Future longitudinal studies incorporating comprehensive assessments of lifestyle and behavioural factors are warranted to better understand these relationships among young adults in rapidly urbanizing settings. Such evidence may help inform context-specific strategies to improve dietary environments and promote healthier choices in similar settings. Approaches such as improving food environments in and around educational institutions, promoting access to healthier food options, and enhancing nutritional awareness among students and their families may be considered as potential areas for intervention, pending stronger evidence from longitudinal studies.

## Figures and Tables

**Table 1:** Distribution of systolic and diastolic blood pressure and prevalence of high blood pressure by socio-demographic and economic characteristics among young adults.

Variable	Category	N	%	Mean, SD/ SBP	Mean, SD/ DBP	High BP n%
Age	≤20	211	68.0	109.5 (15.9)	75.01 (11.5)	20 (9.4)
	>20	100	32.0	116.3 (15.4)	79.14 (10.5)	19 (19.2)
Gender	Male	198	64.0	118.01 (14.0)	79.0 (11.8)	35 (17.6)
	Female	113	36.0	99.76 (12.4)	71.64 (8.7)	4 (3.5)
Type of college	Government	226	73.0	111 (15.6)	76.3 (10.6)	32 (14.2)
	Private	85	27.0	110.1 (17.3)	76.37 (13.2)	7 (8.2)
Place of residency	Home	260	83.6	111.03 (15.9)	76.09 (11.4)	32 (12.3)
	Hostel/PG/other	51	16.4	113.19 (17.0)	77.6 (11.2)	7 (13.7)
Year of study	UG 1 and UG 2	215	68.9	110.0 (16.0)	75 (11.2)	21 (9.7)
	UG 3 and UG 4	96	29.9	114.0 (15.7)	78.9 (11.2)	18 (18.7)
Family income	≤₹100,000	292	93.89	111.0 (14.5)	76.0 (11.3)	33 (11.3)
	>₹100,000	19	6.1	114.6 (14.5)	80.3 (10.5)	6 (31.6)
Father’s occupation	Government employee	33	10.0	112.0 (15.1)	77.1 (9.7)	7 (21.2)
	Self-employed	141	45.3	111.26 (15.1)	75.6 (10.3)	15 (10.6)
	Private employee	94	30.2	111.9 (16.8)	77.4 (13.7)	12 (12.7)
	Other	43	13.5	110.0 (18.0)	75.7 (10.1)	5 (11.60)
Mother’s occupation	Government employee/private employee	47	15.1	106.7 (14.3)	74.1 (9.7)	4 (8.5)
	Home maker	227	72.9	111.9 (16.2)	76.7 (11.6)	29 (12.7)
	Self employed	25	8.0	109.8 (12.9)	75.5 (10.9)	3 (12.0)
	Other	12	3.7	121.1 (21.4)	79.0 (14.8)	3 (25.0)
Father’s education level	Primary	98	31.5	109.7 (15.6)	75 (10.1)	8 (8.2)
	Intermediate	68	21.9	113.0 (14.7)	76.9 (9.8)	9 (13.2)
	Graduate and above	59	19.0	114.6 (18.2)	78.4 (15.6)	10 (16.9)
	No formal education	61	19.6	109.7 (15.0)	75.4 (10.1)	8 (13.1)
	Other	25	8.0	110.2 (19.4)	76.4 (10.5)	4 (10.6)
Mother’s education level	Primary	112	36.0	110.9 (13.7)	76.5 (10.4)	15 (13.0)
	Intermediate	44	14.1	107.0 (15.0)	73.0 (9.0)	1 (2.2)
	Graduate and above	51	16.4	113.0 (17.0)	76.0 (10.9)	10 (19.6)
	No formal education	104	33.4	112.0 (18.0)	77.1 (13.2)	13 (12.5)

Note SBP—systolic blood pressure; DBP—diastolic blood pressure; BP—blood pressure; UG—undergraduate; PG—postgraduate.

**Table 2:** Age- and sex-specific distribution of blood pressure categories among younger adults.

Variable	Category	N	Normal	Elevated (120–129/<80)	High BP Stage 1 (130–139/80–89)	High BP Stage 2 (≥140/≥90)
			n (%) (95% CI)	n (%) (95% CI)	n (%) (95% CI)	n (%) (95% CI)
Age	Below 20 years	212	131 (61.8) (55.1–68.3)	12 (5.7) (3.2–9.4)	49 (23.1) (17.8–29.1)	20 (9.4) (6.1–14.0)
	Above 20 years	99	44 (44.4) (34.9–54.3)	7 (7.1) (3.1–13.5)	29 (29.3) (21.0–38.8)	19 (19.2) (12.3–27.8)
Gender	Male	198	85 (42.9) (36.2–49.9)	17 (8.6) (5.2–13.1)	61 (30.8) (24.6–37.5)	35 (17.7) (12.8–23.5)
	Female	113	90 (79.6) (71.5–86.3)	2 (1.7) (0.3–5.7)	17 (15.1) (9.3–22.5)	4 (3.6) (1.13–8.31)
Overall		311	175 (56.3) (50.7–61.7)	19 (6.1) (3.8–9.2)	78 (25.1) (20.5–30.1)	39 (12.5) (9.2–16.6)

**Table 3:** Association between blood pressure status and ultra-processed food consumption among young adults.

Variable	Category	Normal n%	High BP n%	Total	Chi Square *p* Value
High-Salt Foods	<Median	123 (45.2)	19 (48.7)	142 (45.7)	0.682
	≥Median	149 (54.8)	20 (51.3)	169 (54.3)	
High-Fat Foods	<Median	116 (42.6)	11 (28.2)	127 (40.8)	0.086
	≥Median	156 (57.4)	28 (71.8)	184 (59.2)	
High-Fat and Salt Foods	<Median	127 (46.7)	11 (28.2)	138 (44.37)	0.030
	≥Median	145 (53.3)	28 (71.8)	173 (55.6)	
High-Sugar Foods	<Median	133 (48.9)	15 (38.5)	148 (47.6)	0.222
	≥Median	139 (51.1)	24 (61.5)	163 (52.4)	
High-Sugar and Fat Foods	<Median	136 (50.0)	18 (46.1)	154 (49.5)	0.653
	≥Median	136 (50.0)	21 (53.9)	157 (50.5)	
High-Sugar and Salt Foods	<Median	136 (50.0)	13 (33.3)	149 (47.9)	0.051
	≥Median	136 (50.0)	26 (66.7)	162 (52.1)	
Any High-Fat and/or High-Salt Foods	<Median	134 (49.3)	9 (23.1)	143 (46.0)	0.002
	≥Median	138 (50.7)	30 (76.9)	168 (54.0)	
UPF Diversity Category (Shannon Index)	Low diversity (<50th percentile)	140 (51.5)	15 (38.5)	155 (49.8)	0.129
	High diversity (≥50th percentile)	132 (48.5)	24 (61.5)	156 (50.2)	
UPF Diversity Continuous (Shannon Index)	Median (IQR)	3.01 (2.7–3.2)	3.05 (2.9–3.2)	3.02 (2.7–3.2)	0.150

**Table 4:** Association of ultra-processed food consumption and other covariates with high blood pressure among younger adults using multivariable logistic regression analysis.

Variable	Category	Crude OR (95% CI)	*p* Value	Adjusted OR (95% CI)	*p* Value
Age (years)	≤20	1 (Ref)		1 (Ref)	
	>20	2.28	0.018	1.32 (0.54–3.19)	0.538
Gender	Female	1 (Ref)		1 (Ref)	
	Male	5.85	0.001	4.96 (1.64–15.01)	0.005
Type of college	Private	1 (Ref)		1 (Ref)	
	Government	1.83	0.165	2.10 (0.85–5.22)	0.108
Residency	Home	1 (Ref)		1 (Ref)	
	Hostel/Others	1.13	0.78	0.73 (0.28–1.90)	0.52
Year of study	UG 1–2	1 (Ref)		1 (Ref)	
	UG 3–4	2.13	0.03	1.83 (0.74–4.51)	0.184
Family income	≤₹100,000	1 (Ref)		1 (Ref)	
	>₹100,000	3.62	0.015	3.22 (1.07–9.66)	0.037
High-salt + sugar foods	<Median	1 (Ref)		1 (Ref)	
	≥Median	2.01	0.055	1.25 (0.53–2.91)	0.604
Any high-fat and/or high-salt foods	<Median	1 (Ref)		1 (Ref)	
	≥Median	3.23	0.003	2.85 (1.16–6.99)	0.022

## Data Availability

The data of this study are available from the corresponding author upon reasonable request.

## Supplementary Materials

The following supporting information can be downloaded at: https://www.mdpi.com/article/10.3390/nu18101617/s1. Table S1: Nutrient-Based Classification of Ultra-Processed Foods Included in the Study; Table S2: Univariable logistic regression model for factors associated with high blood pressure; Table S3: Food Frequency Questionnaire (FFQ) for Assessment of Ultra-Processed Food Consumption Among Young Adults; Figure S1: Relationship between ultra-processed food (UPF) score and systolic and diastolic blood pressure among young adults.

## Abbreviations

AHA	American Heart Association
BP	Blood pressure
CVD	Cardiovascular disease
DBP	Diastolic blood pressure
FFQ	Food frequency questionnaire
IFCTs	Indian Food Composition Tables
LMICs	Low- and middle-income countries
PG	Post-graduate
SBP	Systolic blood pressure
UG	Undergraduate
UPF	Ultra-processed food
WHO	World Health Organization
DALYs	Disability-adjusted life years
