# Supplementary material for: Ultra-Processed Food Consumption Patterns and Their Association with Blood Pressure Among Young Adults: A Cross-Sectional Study
Source: Nutrients. 2026 May 20;18(10):1617. doi: 10.3390/nu18101617 (PMC13209713; doi:10.3390/nu18101617)
Supplement: Supplementary file 1 [file nutrients-18-01617-s001.zip › nutrients-4246626-supplementary.pdf]

Supplementary Table.S1: **Nutrient-Based Classification of Ultra-Processed Foods Included in the Study**

**Food item**	**Category**	**Na** (mg/100g)	**Sugar** (mg/100g)	**Fat** (mg/100g)	**Energy** (kcal/100g)
Biscuits	High Sugar + High Fat	370	21.16	10.58	406
Bread	High Sugar + High Fat	477	5.34	3.59	267
Packed breakfast cereals (Oats, cornflakes, muesli)	High Sugar + High Fat	209.8	10.86	5.2	390.3
Bun	High Sugar + High Fat	450	5.34	3.59	267
Cakes/cake mixes	High Sugar + High Fat	304	39.53	14.77	352
Carbonated beverages	High Sugar	13	9.9	0.25	45
Cheese	High Fat + High Salt	1220	1.5	25	311
Chocolates	High Sugar + High Fat	129.3	57.3	28.7	529
Cookies	High Sugar + High Fat	311	32.9	24.72	492
Energy drinks	High Sugar	41	11	0	45
Energy/Protein bars	High Sugar + High Fat	238	28.75	12.5	415
Flavoured yogurt	High Sugar	59	17	1.3	94
Frozen foods with additives	High Fat	478	1.31	11.12	250
Tetra pack Fruit juices	High Sugar	34	12	0	50
Ice creams	High Sugar + High Fat	90	22.86	11.3	221
Jams	High Sugar	26	49.16	0.03	261
Instant noodles	High Fat + High Salt	1123.5	1.7	16.5	421.5
Mayonnaise	High Fat + High Salt	635	0.57	74.85	680
Other Instant foods	High Salt	905	2.9	3.1	286
Pastries	High Sugar + High Fat	304	39.53	14.77	352
Sauces	High Sugar + High Salt	880	34	0.4	159.3
Savoury/sweet packaged foods	High Fat	587	0.81	36.5	558
Spreads	High Sugar + High Fat	377	30.9	35.8	508.1
Protein powders	Below thresholds	156	0.0	1.6	352

Nutrient values (per 100 g) were obtained from packaged food labels and supplemented, where necessary, with data from the USDA Food Composition Database. Classification of ultra-processed foods into high sugar, high salt, high fat, and combined categories was based on threshold criteria defined in the Indian Dietary Guidelines (DGI).

Supplementary Figure S1. Relationship between ultra-processed food (UPF) score and systolic and diastolic blood pressure among young adults

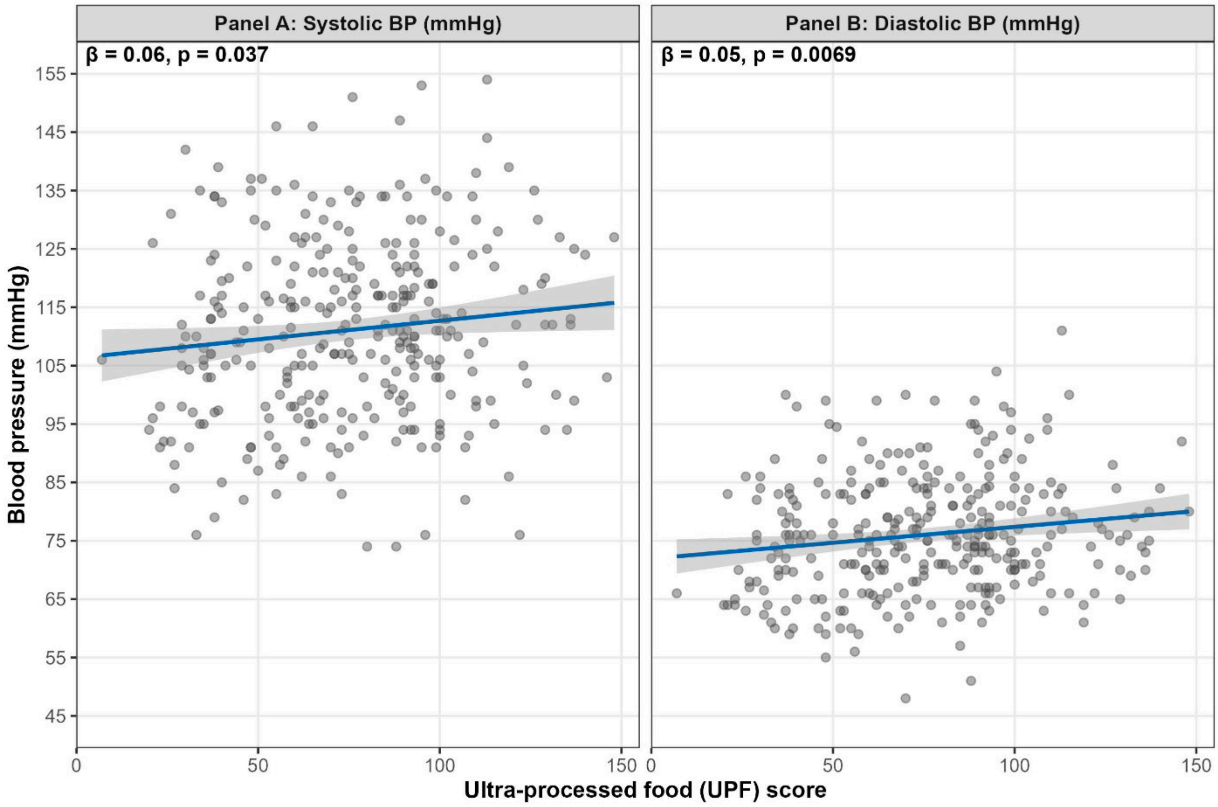

Supplementary Fig.1 :Panel A shows the relationship between UPF score and SBP, Panel B depicts the association with DBP. Each point represents an individual participant, and the solid line indicates the fitted linear regression with the shaded area representing the 95% confidence interval.

Supplementary Table S2 : Univariable logistic regression model for factors associated with high blood pressure.

**Variable**	**Category**	**Crude OR**	**P value**
Age (Yrs)	<=20	1	
	>20	2.28	0.018
Gender	Female	1	
	Male	5.85	0.001
Type of college	Private	1	
	Government	1.83	0.165
Place of residency	Home	1	
	Hostel/PG/Others	1.13	0.780
Year of Study	UG 1 and 2	1	
	UG 3 and 4	2.13	0.030
Family Income	<10,000-	1	
	> ₹1,00,000	3.62	0.015
Father's education	Not going to school	1	-
	Primary	0.58	0.317
	Intermediate	1.01	0.984
	Graduate and above	1.35	0.557
	Other	1.26	0.726
Father Occupation	Govt employee	1	
	Private employee	0.54	0.247
	Self employed	0.44	0.107
	Others	0.49	0.262
Mother education	Not going to school	1	
	Primary	1.08	0.246
	Intermediate	0.16	0.085
	Graduate and above	1.70	0.246
Mother occupation	Govt/Private employee	1	
	Home maker	1.57	0.417
	Self employed	1.46	0.636
	Others	3.58	0.132
High Salt Foods	< Median	1	
	>=Median	0.87	0.682
High Fat Foods	< Median	1	
	>=Median	1.89	0.090
High Fat and Salt Foods	< Median	1	
	>=Median	2.22	0.033
High Sugar Foods	< Median	1	
	>=Median	1.53	0.225
High Sugar and Fat Foods	< Median	1	
	>=Median	1.16	0.653
High Salt and Sugar	< Median	1	
	>=Median	2.01	0.055
Any High Fat and/or High Salt Foods	< Median	1	
	>=Median	3.23	0.003
UPF diversity category (Shannon index)	Low diversity (<50th percentile)	1	
	High diversity (≥50th percentile)	1.70	0.132

Supplementary Table S3: Food Frequency Questionnaire (FFQ) for Assessment of Ultra-Processed Food Consumption Among Young Adults

Indicate how often you consume the following food items	Daily	Thrice a week	Twice a week	Once a week	Once in 15 days	Once a month	Rarely	Never
Biscuits								
Bread								
Packed breakfast cereals (Oats, cornflakes, muesli)								
Bun								
Cakes/cake mixes								
Carbonated beverages								
Cheese								
Chocolates								
Cookies								
Energy drinks								
Energy/Protein bars								
Flavoured yogurt								
Frozen foods with additives								
Tetra pack Fruit juices								
Ice creams								
Jams								
Instant noodles								
Mayonnaise								
Other Instant foods								
Pastries								
Protein powders								
Sauces								
Savoury/sweet packaged foods								
Spreads								
